# Malevich, an hero pushed to death

**DOI:** 10.1080/17571472.2015.1133951

**Published:** 2016-02-24

**Authors:** Francesco Carelli

**Affiliations:** ^a^ Professor Family Medicine, Milan, Rome; ^b^ EURACT Council Executive

**Keywords:** advangarde, leader, banning, hero

Kazimir Malevich (1878–1935) was a radical, mysterious and hugely influential figure in modern art. He lived and worked through one of the most turbulent periods in twentieth-century history. In 2014, Tate Modern in London presented the first major Malevich retrospective for almost twenty-five years. This groundbreaking exhibition drew on the world’s greatest collections of his work to offer an expansive view of his career.

Having come of age in Tsarist Russia, Malevich witnessed the October Revolution first-hand. His early experiments as a painter led him towards the cataclysmic invention of *Suprematism*, a bold visual language of abstract geometric shapes and stark colours, epitomised by his piece: *Black Square*. A definitively radical gesture, it was revealed to the world after months of secrecy and was hidden again for almost half a century after its creator’s death. It sits on a par with Duchamp’s ‘ready-mades’ as a game-changing moment in twentieth-century art and continues to inspire and confound viewers to this day.

The show explored his collaborative involvement with architecture and theatre, including his designs for the avant-garde opera *Victory over the Sun*. The exhibition also followed his temporary abandonment of painting in favour of teaching and writing, and his much-debated return to figurative painting in later life.

Malevich’s work tells a fascinating story about the dream of a new social order, the successes and pitfalls of revolutionary ideals, and the power of art itself. This exhibition, for the first time, offered a chance to trace his groundbreaking developments not only through well-known masterpieces but also through earlier and later work, sculpture, design objects, and rarely seen prints and drawings.

## Death of an hero

Malevich’s body placed into Suetin’s coffin, shortly after the artist’s death, 1935.

**Figure 1.**
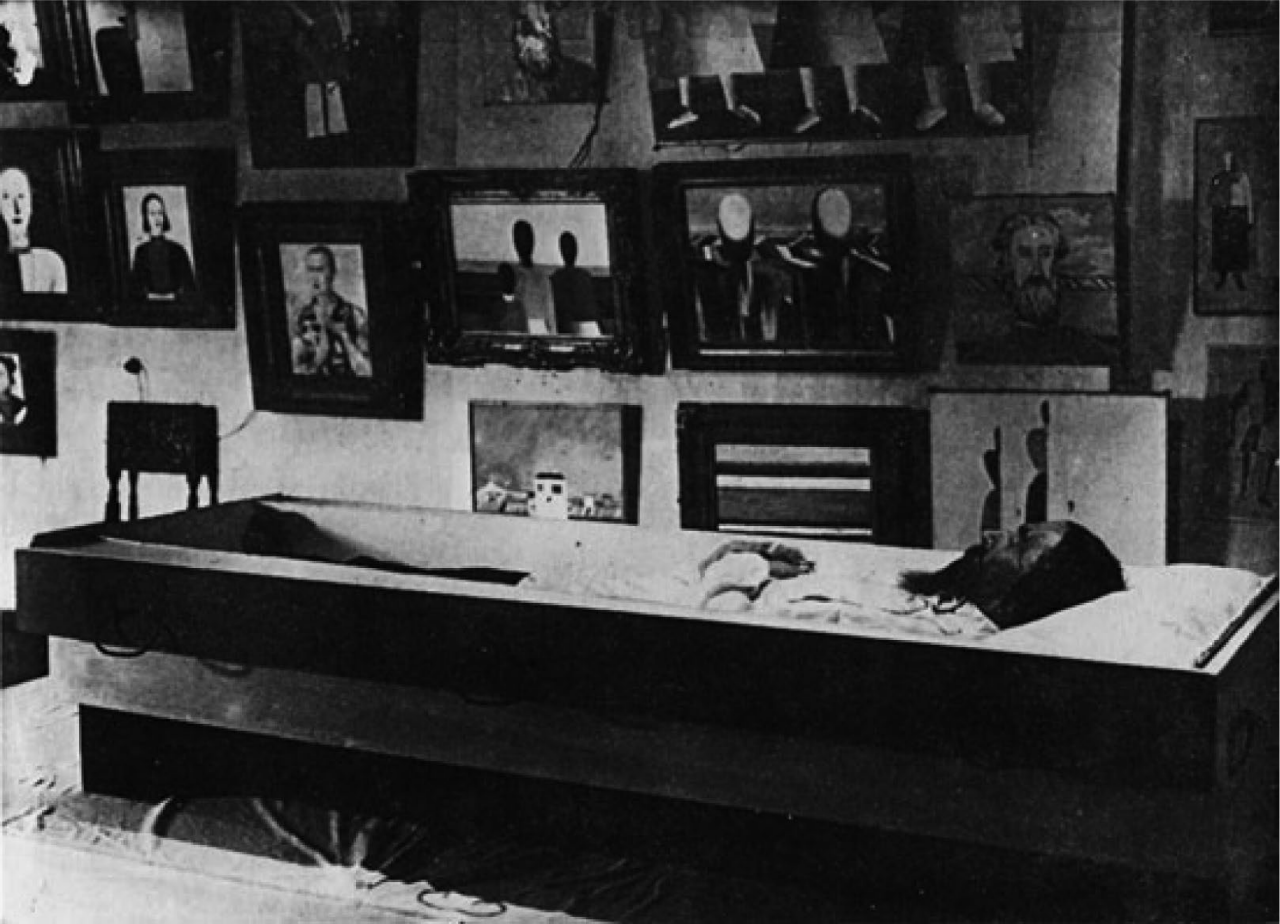
Malevich’s body placed into Suetin’s coffin, shortly after the artist’s death, 1935. Source: Tate Modern.

Malevich, in his struggle with a banal daily existence, was under constant criticism from opponents in both the artistic community and the Soviet regime. He met with open reprisals following his return from his sole foreign trip to Poland and Germany in 1927. These included unfair and thoughtless administrative decisions as well as actions by the security services, undertaken until the last days of his life.

Even his very name was banned, though some were aware of the persecuted artist’s greatness and tried to protect him, seeking out ways to circumvent regulations in the name of higher values. Still, they were obliged to follow orders from ‘above’ (the KGB). The truth is bitter and dramatic. It spans virtually the whole of his life, with short, promising intermissions. From the very birth of Bolshevik Russia in 1917 – there was a more or less brutal smear campaign against the artist. This was both open and covert, via the press and artistic organisations, and subordinated to the party’s directives.

He was arrested several times , firstly in Vitebsk (he was saved then by a letter from Lunacharsky they found on him). He was accused of involvement in subversive, anti-state activities and suffered oppressive, humiliating censorship from the local authorities (1919–1921)which drove the beginning of starvation-induced tuberculosis.

Being under constant surveillance from secret agents or planted provocateurs (a mixture of truth and fabrication) he lived in poverty with sporadic purchases of his works by museums or collectors.

Extremely prolific correspondence reveals that his second wife, Zofia fell ill with tuberculosis, lacking money for medication. During his experiments and his teaching (at the Artistic Culture Institute, INChUK), he was harassed by constant inspections, with the real threat of the unique institution’s liquidation.

Eventually the Institute was closed and his personal freedom was restricted to a minimum; and another arrest led to suspicions of spying and engaging in counter-revolutionary activities while abroad. In jail he was tortured. This caused even more acute poverty, and near starvation.

Following the liquidation of the Artistic Culture Institute, some Soviet institutions moved into its premises. The artist and his family were allowed to remain in the three-room flat No. 5, but the front door was walled up, cutting the residents off from the main staircase. The door connecting the apartment with what used to be the Institute’s premises remained in place, like a stage prop. Behind it was a wall. That left only the back door, the servants’ entrance, or ‘ black entrance’. This was a deliberate step to humiliate the artist, who was then still artistically active.

His apartment and studio were very cold and he lacked proper shoes and warm clothes. He developed cancer and his doctors recomended therapy abroad in Paris, to be paid for with proceeds from the sale of paintings. However his passport application was rejected leading to a de facto house arrest.

The organs of state were well aware of Malevich’s critical health condition, but did nothing to save a world-renowned artist. This suggests that the Lubyanka was waiting for a natural solution – by his death. And so it happened.

Was it a deliberate strategy, a quasi criminal method to get rid of an aesthetic dissident? We know now of one of the KGB officials’ recorded words: *Don’t touch the old man, do nothing, wait, let it happen by itself.*

The closest family, his mother Ludwika, his wife Natalia, his daughter Una and also his disciples, Leporskaya, Rozhdestvensky and Suetin: all were helpless by the sick bed. His eyes in the photographs say that the end is near. We know the details of Malevich’s original design of his own coffin (executed by Suetin). It was an elongated, suprematist shape with offsets and profiles. Suprematist symbols were on the lid: a square, a circle. His final expressive gesture, his message for the world,was indomitable even then.

Malevich had also asked for his body to be given a vertical and horizontal form – arms spread. He himself, his body, was to play the role of a sign, a *supremus*. The narrow entrance door, stairs, backyard staircase made any complicated manoeuvres with the catafalque impossible, threatening to violate the body’s integrity: everyone present was aware of that.

The photograph showing the catafalque being taken outside through the back door, carried by Suetin and Rozhdestvensky, and bearing the legend *‘The catafalque being escorted from the Artists’ House’*. They had to remove the body from the coffin to be able to negotiate the latter through this door. When they were escorting the coffin from the apartment, they had to put it upright because the stairs were so narrow.

A plaque with the ‘black square’ was affixed to the rectangular coffin and an appropriate plate was placed on a nearby tree. Soon the symbolic structure with the suprematist ‘black square’ disappeared. The field overgrew with weeds and after the war it was ploughed. Some hand removed that piece of evidence too.

A 1918 fragment of one of Malevich’s poems seems apposite here:

*I part with the earth, currents crossing my consciousness reason increasing. We move beyond the horizon to break free of the earth scattering in the cup*-*dome of space like scattered points* (1918)

The last chapter of Malevich’s life was an experience of humiliations*,* he being an islet of independent thought in an ocean of totalitarism which was horribly brutal and omnipresent. Independent-minded artists all suffered this kind of ruthlessness. After his death too the public presentation of his works in the Soviet Union was banned

After nearly 80 years since Kazimir Malevich’s death, knowledge about this prematurely deceased pioneer of 20th-century art has become incomparably more extensive than before. This is thanks, in large part, to the efforts of contemporary Russian historians, archivists and publishers.

